# Mitochondrial Ca^2+^ Homeostasis: Emerging Roles and Clinical Significance in Cardiac Remodeling

**DOI:** 10.3390/ijms23063025

**Published:** 2022-03-11

**Authors:** Dejiu Zhang, Fei Wang, Peifeng Li, Yanyan Gao

**Affiliations:** Institute for Translational Medicine, College of Medicine, Qingdao University, Qingdao 266021, China; dejiuzhang@hotmail.com (D.Z.); wangfeea@163.com (F.W.); peifli@qdu.edu.cn (P.L.)

**Keywords:** mitochondria, Ca^2+^ homeostasis, cardiac remodeling, mitochondrial Ca^2+^ uniporter protein complex, cardiovascular diseases

## Abstract

Mitochondria are the sites of oxidative metabolism in eukaryotes where the metabolites of sugars, fats, and amino acids are oxidized to harvest energy. Notably, mitochondria store Ca^2+^ and work in synergy with organelles such as the endoplasmic reticulum and extracellular matrix to control the dynamic balance of Ca^2+^ concentration in cells. Mitochondria are the vital organelles in heart tissue. Mitochondrial Ca^2+^ homeostasis is particularly important for maintaining the physiological and pathological mechanisms of the heart. Mitochondrial Ca^2+^ homeostasis plays a key role in the regulation of cardiac energy metabolism, mechanisms of death, oxygen free radical production, and autophagy. The imbalance of mitochondrial Ca^2+^ balance is closely associated with cardiac remodeling. The mitochondrial Ca^2+^ uniporter (mtCU) protein complex is responsible for the uptake and release of mitochondrial Ca^2+^ and regulation of Ca^2+^ homeostasis in mitochondria and consequently, in cells. This review summarizes the mechanisms of mitochondrial Ca^2+^ homeostasis in physiological and pathological cardiac remodeling and the regulatory effects of the mitochondrial calcium regulatory complex on cardiac energy metabolism, cell death, and autophagy, and also provides the theoretical basis for mitochondrial Ca^2+^ as a novel target for the treatment of cardiovascular diseases.

## 1. Introduction

Mitochondria are called “power stations” because it is here that cells perform aerobic respiration and produce energy. In addition to generating energy for cells, mitochondria also participate in apoptosis, the tricarboxylic acid cycle, Ca^2+^ signal transduction, information transmission, and other processes, and also regulate cell growth and cell cycle [1].

The heart is the largest energy-consumption organ and mitochondria are its main source of energy [2]. Unlike non-cardiac mitochondria, adult cardiac mitochondria are partially immobile and their ability to move and distribute in the cytoplasmic tubular network is limited [3]. According to the location and function of mitochondria in adult cardiomyocytes, they can be divided into the following three categories: interfibrillar mitochondria (IFM), subsarcolemmal mitochondria (SSM), and perinuclear mitochondria [4]. IFM and SSM are distinct physiological types located at different regions of cardiac tissue [4,5]. IFM is mainly of a tubular structure, which is aligned longitudinally between myofibrils. Earlier studies have shown that IFM has a higher substrate oxidation rate (about 1.5 times) than SSM. Therefore, IFM located in myofibrils is thought to provide a large amount of energy for myocardial cell contraction [6]. Their tubular cristae are involved in ATP production for cardiac contractions and Ca^2+^ signaling [6]. SSM with a lamelliform structure is positioned mainly beneath the subsarcolemmal [7]. Under normal conditions, mitochondria produce ATP through oxidative phosphorylation, which provides energy for the normal contraction and metabolism of cardiomyocytes, and maintains cellular homeostasis [2]. The pathophysiology of cardiomyocytes is associated with changes in mitochondria, including swelling, loss or reorientation of cristae, structural deformation, or internal and external ventricular vacuoles [8]. So the stability of mitochondrial morphology and function is particularly important for the maintenance of normal cardiac physiological function [7].

Mitochondrial homeostasis refers to the mechanism that maintains the integrity of the mitochondrial genome and proteome and the normal function of mitochondria [9]. Mitochondrial Ca^2+^ homeostasis is an important aspect of mitochondrial homeostasis. Mitochondrial Ca^2+^ homeostasis plays a series of key roles in cell physiological and pathological processes, including energy metabolism, apoptosis, and the production of reactive oxygen species (ROS) [10]. Mitochondrial Ca^2+^ exchange, that is, Ca^2+^ flowing in and out of mitochondria, is the most fundamental factor for balancing cell death and energy demand. The imbalance of mitochondrial Ca^2+^ homeostasis plays a key role in the occurrence and development of cardiovascular diseases [11,12]. Mitochondrial Ca^2+^ channels are a promising therapeutic target for alleviating irreversible and severe symptoms of cardiac dysfunction [13]. Targeting mitochondrial Ca^2+^ homeostasis provides new therapeutic strategies for aging-related diseases, particularly cardiovascular diseases [14].

Under physiological and pathophysiological conditions, intracellular Ca^2+^ plays an important role in regulating excitation–contraction (EC) coupling, cell proliferation and differentiation, and the cell death of cardiomyocytes. Cytoplasmic Ca^2+^ ([Ca^2+^] cyto) affects a variety of other targets, including ion channels and transporters, signaling cascades, gene transcription, and mitochondrial ATP production [15,16]. However, Ca^2+^ is not only a key element in EC-coupling, but also a key second messenger in cardiac signal transduction, controlling excitatory, metabolic, and transcriptional processes [17]. Intracellular Ca^2+^ fluctuations that activate contractile apparatus promote the excitation–contractile coupling of cardiomyocytes. During electrical stimulation, the amount of Ca^2+^ released from the sarcoplasmic reticulum (SR) into the cytoplasm regulates the formation of cross-bridges between myofilaments, thus determining the force generated by the myocardium. The diastolic phase promotes the dissociation of Ca^2+^ from troponin C, and cytosolic Ca^2+^ clearance determines the pattern of muscle relaxation. Therefore, defects in intracellular Ca^2+^ processing may be the cause of impaired systolic and diastolic function in hearts. In adult myocardium, the sarcoplasmic reticulum (SR) is the main source and reservoir of cytoplasmic Ca^2+^. SR regulates Ca^2+^ release through Ca^2+^ release channels or type 2 Ryanodine receptor (RyR2), and is essential for the excitation–contraction (EC) coupling of cardiomyocytes through the induced Ca^2+^ release (CICR) mechanism [18,19]. The interruption of Ca^2+^ treatment can lead to the pathogenesis of many diseases, such as Alzheimer’s disease, Huntington’s disease, and congestive heart failure [20]. Mitochondrial Ca^2+^ regulates different processes that are crucial to cellular function, such as energy production (ATP), mitochondrial permeability transition pore (mPTP) opening, triggering, and preventing apoptosis [21]. There are several potential Ca^2+^ influx and outflow sites in mitochondria. The concentration of Ca^2+^ in mitochondria depends on the pathways across the endoplasmic reticulum, mitochondria-associated membranes (MAMs), and mitochondria [22]. It has long been recognized that Ca^2+^ signaling in mitochondria not only regulates mitochondrial metabolism but also promotes cell death. However, under cardiac ischemia-reperfusion injury and other pathological conditions, cytoplasmic Ca^2+^ overload prevents mitochondrial Ca^2+^ from upregulating mitochondrial ATP production and promotes the mitochondrial death pathway [23,24]. In addition, mitochondrial Ca^2+^ activates TCA cycle dehydrogenase and regulates nicotinamide adenine dinucleotide (NADH) production, affecting the antioxidant capacity of cells and the production of mitochondrial ROS, thereby playing an important role in regulating the redox state of cells [25]. Therefore, the mitochondrial Ca^2+^ pathway plays an important role in the regulation of cellular functions and the cell death pathway. We believe that mitochondrial Ca^2+^ homeostasis is a double-edged sword regulating cardiac mitochondrial function. Although mitochondria Ca^2+^ overload prevention has attractive therapeutic potential, a wide range of diseases, and no entity for mitochondrial Ca^2+^ exchange into clinical trials [26]. This review provides an overview of mitochondrial Ca^2+^ homeostasis in regulating cardiac energy metabolism, cell death, ROS production and autophagy. As well as the mechanism of mitochondrial Ca^2+^ homeostasis regulation in physiological and pathological heart remodeling, it provides a theoretical basis for mitochondrial Ca^2+^ as a new therapeutic target for cardiovascular diseases and clinical treatment.

## 2. Mitochondrial Ca^2+^ Homeostasis and Cardiac Energy Regulation

In the heart muscle tissue, the action potential (AP) activates voltage-gated Na^+^ channels and induces the rapid depolarization of the cell membrane, facilitating the voltage-dependent opening of L-type Ca^2+^ channels and Ca^2+^ entry into the cells. With a Ca^2+^ influx, the sarcoplasmic reticulum (SR) Ryanodine Type 2 (RyR2) channel is activated, resulting in a large amount of Ca^2+^ release from SR, leading to a transient increase in cytosolic Ca^2+^ and the activation of myofilament cross-bridge formation. At the end of contraction, Ca^2+^ enters the SR through SR Ca^2+^-ATPase (SERCA) and flows out of the extracellular space through the Na^+^/Ca^2+^ exchanger (NCX) [27,28].

Furthermore, SERCA1, 2, and 3 are variable splicing isoforms of the SR Ca^2+^-ATPase gene. In cardiac tissue, the Ca^2+^-ATPase (SERCA2a) isoforms promote Ca^2+^ storage and distribution in SR. The SERCA2a of the sarcoplasmic reticulum (SR) maintains a 1000-fold Ca^2+^ gradient on the cardiac sarcoplasmic reticulum and plays a dominant role in the excitation–contraction coupling and contractility of the heart. During systole, action potentials induce a small amount of Ca^2+^ to flow through L-type Ca^2+^ channels from the sarcolemma. This influx initiates Ca^2+^ releasing channels or Ryanodine receptors (RyR) to release Ca^2+^ in large quantities from SR Ca^2+^ stores [29]. During diastole, Ca^2+^ entering the SR or extracellular lumen is quickly removed. This process is promoted mainly by SERCA (70–80% Ca^2+^ removal in higher mammalian and human myocardium) and a small amount by sarcolemmal Na^+^, Ca^2+^-exchange (20–30%) and a slower Ca^2+^-transport system [30]. The Ca^2+^ in SR binds mainly to the SR Ca^2+^ binding proteins calsequestrin and calreticulin and histidine-rich binding proteins [31]. As is known, Ca^2+^ is stored in the vicinity of Ca^2+^ release channels via the proteins triadin and Junctin [32,33], which is likely due to the accelerated availability of Ca^2+^ near ryanodine receptors during early contraction. In addition to SERCA2a, the phosphorylation status of the ryanodine receptor and its accessory proteins may modulate Ca^2+^ release at the SR level [34].

**Figure 1:**
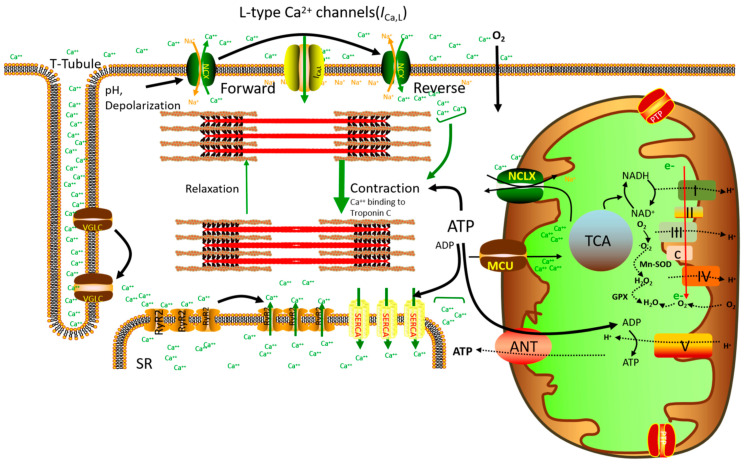
Under physiological conditions, Ca^2+^ regulates excitation–contraction coupling, mitochondrial energetics, and ROS production in cardiomyocytes. In cardiomyocytes, action potential causes Ca^2+^ to enter cells from L-type Ca^2+^ channels (Ica, L), and the influx of Ca^2+^ activates Ryanodine Type 2 (RyR2) on the sarcoplasmic reticulum (SR), resulting in a large release of Ca^2+^ from the SR and subsequent binding to troponin C promoting myofilament cross-bridge formation, which causes cardiac contraction. During systole, Ca^2+^ enters SR via SR Ca^2+^-ATPase (SERCA) and exits the extracellular space via the Na^+^/Ca^2+^ exchanger (NCX). Mitochondria take up Ca^2+^ through MCU, and Ca^2+^ activate two key enzymes, isocratic dehydrogenase, and α-ketoglutarate dehydrogenase, of the TCA cycle and regenerate NADH^+^ from NAD. This causes electrons to move along the electron transfer chain (ETC) from complex I to complex IV. Complexes I, III, and IV pump protons (H^+^) into the intermembrane space, forming proton-motive forces bearing electrochemical potential and a proton gradient. Compound V converts ADP to ATP under proton drive. ATP is released into the cytoplasm through the adenine nucleoside transporters (ANT) on the inner membrane of mitochondria.

Mitochondria occupy more than 30% of the heart cellular volume and occur close to the main energy-consuming sites, that is, the myofilaments, SR, and t-tubules. Mitochondria are the heart’s energy factories, providing more than 90% of ATP for cardiac contraction [35,36]. Mitochondria produce energy through oxidative phosphorylation, which is consumed by cardiac excitation–contraction (EC) coupling. Mitochondria play an important role in cardiac physiology and pathophysiology, and Ca^2+^ is at the core of cardiac EC coupling [28]. In chronic heart failure, EC disorder may adversely affect mitochondrial Ca^2+^ uptake and energy production, resulting in a vicious circle of cardiac systolic dysfunction and energy loss [37]. It has been shown in cell models that mitochondria regulate the TCA cycle and increase the activity of the electron transfer chain (ETC) to promote ATP production through Ca^2+^ uptake [38]. Mitochondrial oxidative phosphorylation synthesizes ATP through a Ca^2+^-dependent process, so the maintenance of mitochondrial Ca^2+^ homeostasis is crucial for the regulation of mitochondrial ATP production [39]. Under the electrochemical gradient produced by strong Ca^2+^ influx, mitochondria primarily uptake Ca^2+^ through the mitochondrial Ca^2+^ monomolecular carrier (MCU) [40,41]. Mitochondrial Ca^2+^ activates three key enzymes in the TCA cycle, of which, isocratic dehydrogenase and α-ketoglutarate dehydrogenase are activated in a Ca^2+^-dependent manner [38,42,43,44,45,46]. Territo and Balaban found that Ca^2+^ also activated the F1/F0 ATPase [47,48], and increased respiration in less than 100 ms, a rate sufficient to support the conversion of cardiac function in vivo. This triggers an increase in the conversion of nicotinamide adenine dinucleotide (NAD^+^) to reduced NADH, moving electrons along the ETC from complex I to complex IV. Protons (H^+^) are pumped into the intermembrane space by complexes I, III, and IV, establishing a proton motive force via electrochemical potential and a proton gradient. Compound V is driven by this proton motive force to convert ADP into ATP [47,48,49]. ATP is then released into the cytoplasm by adenine nucleoside transporter (ANT) on the inner membrane of mitochondria and voltage-dependent anion channel (VDAC) on the outer membrane of mitochondria [50,51,52] (Figure 1). High phosphate buffer systems, such as creatine kinase (CK) isoenzymes and highly diffused phosphocreatine (PCr), exist in the cytoplasm and limit a large number of ATP changes while ADP shuttles from ANT and effectively transfers energy signals from the ATP hydrolysis site to mitochondria. In addition, in complexes I and III, some electrons leak out of the ETC and react with oxygen to form superoxides. In other words, mitochondrial ROS production is also a Ca^2+^ dependent process [53,54,55]. In conclusion, mitochondrial Ca^2+^ homeostasis plays an important role in regulating ATP production and ROS generation [39]. Mitochondria are the main sources of ATP and ROS, and their functioning is strictly controlled by mitochondrial Ca^2+^. In the physiological process of workload, the uptake of mitochondrial Ca^2+^ needs to match the balance of energy supply and demand while maintaining the antioxidant capacity in a reduced state to prevent excessive ROS [56].

In addition to ATP production by the activation of TCA cyclase and ATP synthase, mitochondrial Ca^2+^ has been reported to directly activate L-type Ca^2+^ channels in adult guinea pigs and mouse vascular myocytes, increasing NADH production, oxygen consumption, and ROS production [57,58,59]. Therefore, a change in L-type Ca^2+^ activity may play an important role in Ca^2+^-dependent mitochondrial energetics, but the precise mechanism underlying his phenomenon remains to be elucidated. It has also been reported that the activation of L-type Ca^2+^ channels not only regulates Ca^2+^ influx but also Ψm independently of mitochondrial Ca^2+^ uptake [59]. Mitochondrial Ca^2+^ plays a dual role in the process of energy supply and demand matching of cardiomyocytes. L-shaped Ca^2+^ channels can lead to a Ca^2+^ influx, triggering the release of large amounts of Ca^2+^ from the SR during cardiac systolic and diastolic coupling. Furthermore, Ca^2+^ binds to troponin C and thereby induces cardiac contraction. During the diastolic phase, Ca^2+^ is transported from SERCA to SR, or through the NCX to the extracellular membrane [60]. β-adrenergic stimulation increases the rate and amplitude of cytosolic Ca^2+^ transient. ATP is hydrolyzed to ADP, which then enters mitochondria through the ANT to activate the F1F0-ATPase and regenerate ATP. This accelerates electron flow in the ETC, and NADH is oxidized to NAD+. This is called the “pull” condition. At the same time, MCU uptakes Ca^2+^ into the mitochondria, activates key enzymes of the TCA cycle, and converts NAD+ into NADH. This is called the “push” condition (Figure 1). Therefore, mitochondrial Ca^2+^ can not only pull electrons along the ETC to increase energy consumption but also push electrons from the TCA cycle into the ETC to regenerate energy; this is known as “parallel activation” [47,61,62].

## 3. Mitochondrial Ca^2+^ Homeostasis and the Regulation of Cardiac Cell Death

**Figure 2:**
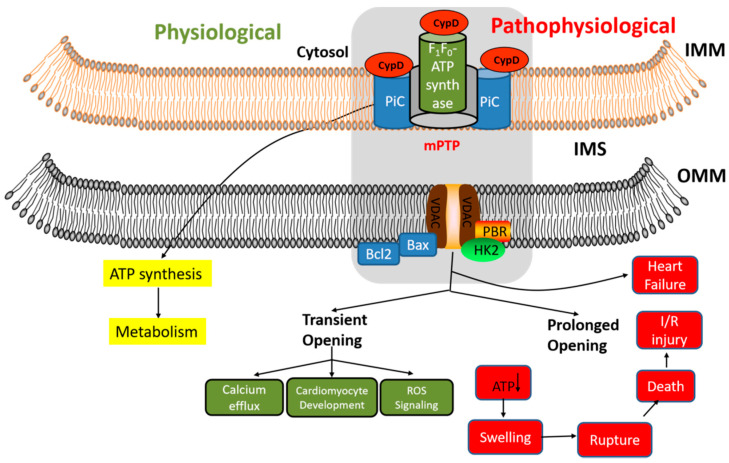
Dual role of mPTP in cardiac physiology and pathology. At the mitochondrial level, mPTP plays a dual role and participates in important physiological processes (ATP production and mitochondrial metabolism) and pathophysiological processes (cardiomyocyte death, heart failure, and I/R injury), while mCa^2+^ overload leads to the opening of mPTP channels. mPTP is a large nonspecific pore of cardiomyocytes. It is a protein complex composed of many proteins, including F1F0-ATP synthase, cyclophilin D(CypD), the phosphate carrier (PiC) and voltage-dependent anion channel (VDAC) among others, that opens through the inner and outer membranes of mitochondria. VDAC is located in the outer membrane of mitochondria. The prolonged opening of mPTP leads to a reduction in ATP production, depolarization of the mitochondrial inner membrane, matrix swelling, rupture of the mitochondrial outer membrane, and cell death. Finally, it causes myocardial ischemia-reperfusion injury and heart failure. The transient opening of the mPTP channel causes calcium efflux, cardiomyocyte development, and ROS signal. IMS, intermembrane space; OMM, outer mitochondrial membrane; IMM, mitochondrial inner membrane. HK2, hexokinase 2; PBR, peripheral benzodiazepine receptor; I/R injury, ischemia-reperfusion injury.

As mentioned above, mitochondrial Ca^2+^ not only regulates mitochondrial energy but also promotes cell necrosis in case of Ca^2+^ overload. A common cause of necrosis is an impaired mitochondrial energy metabolism, resulting in a sharp decline in ATP levels. Necrosis plays an important role in many pathological conditions, including ischemia/reperfusion injury, trauma, and neurodegenerative diseases. Thus, the maintenance of mitochondrial Ca^2+^ homeostasis is of critical importance. Studies have shown that the inhibition of mitochondrial Ca^2+^ uptake can significantly reduce cell death [63]. The history of Ca^2+^-induced cell death can be traced back to Fleckenstein’s study in 1974. He found that excessive Ca^2+^ entry into cells may be the cause of death after cardiac ischemia [64]. Under physiological conditions, the inner membrane of mitochondria is impermeable, but under certain conditions, Ca^2+^ accumulation and oxidative stress in mitochondria can trigger the opening of highly conductive pores in the mitochondrial intermembrane. This phenomenon, known as mitochondrial permeability transition (MPT), leads to changes in mitochondrial morphology and function. MPT is a Ca^2+^ dependent process and is regulated by factors, such as inorganic phosphorus, ATP deficiency, low pH, and oxidative stress (e.g., ROS, oxidized GSH, and pyridine nucleotide pools) [65,66,67]. MPT is followed by mitochondrial osmotic swelling, membrane rupture, and the release of cytochrome c and other mitochondrial proteins into the cytoplasm. During electron transfer, an electrochemical gradient is established that promotes ATP synthase to produce ATP. However, under pathological conditions, mitochondrial Ca^2+^ overload causes the opening of the mPTP, allowing molecules of less than 1.5 kDa to pass freely [23,24]. This results in the disruption of the mitochondrial membrane potential, alteration of membrane permeability, reduction in ATP synthesis, mitochondrial rupture, loss of matrix solute (including GSH, pyridine nucleotides, and ADP/ATP), and the release of cytochrome C from the intermembrane space [24] (Figure 2). Several studies reinforce that an mPTP opening caused by mitochondrial Ca^2+^ overload is a key cause of cardiomyocyte death in ischemia-reperfusion injury [24]. Moreover, the opening of mPTP and the subsequent uncoupling of mitochondria leads to the active hydrolysis of cytosolic ATP and a decrease in ATP content in the cytoplasm, resulting in the disturbance of intracellular Ca^2+^ homeostasis, the activation of various catabolic enzymes (protease, phospholipase, etc.), and cell death. The use of drugs to inhibit or knockout mPTP components holds great promise for preventing cardiomyocyte death. Although the mPTP opening is primarily associated with the necrosis of cells, several cytotoxic drugs have been shown to mediate apoptosis through Ca^2+^-mediated MPT. A persistent PTP opening can be detrimental to mitochondrial function, but a transient opening or flickering of PTP is observed in many cell types [68,69] and isolated mitochondria [70]. The frequency of transient PTP opening was primarily determined by free matrix Ca^2+^ [71,72]. Physiological PTP flicker is considered to be the mechanism of Ca^2+^ release from overloaded mitochondria [70,73,74,75]. In this way, PTP flicker can be used as a physiological safety valve to prevent Ca^2+^ overload, mitochondrial failure, and thereby, cell death (Figure 2). Studies have shown that the inhibition of PTP opening by cyclosporine A (CsA) inhibited mitochondrial Ca^2+^ release from mitochondria in rat cardiocytes [76].

Importantly, Ca^2+^ not only plays a key role in the regulation of cell death, but is also a critical sensitizing signal in mitochondrial pro-apoptotic transition [77]. Mitochondria are important checkpoints in the process of apoptosis, and they activate the internal pathways of apoptosis by releasing cytochrome C and other mitochondrial proteins into the cytosol. Mitochondrial Ca^2+^ overload is also one of the pro-apoptotic pathways, as it is known for inducing mitochondrial swelling, rupturing the outer membrane, and then releasing mitochondrial apoptosis factors into the cytosol [78]. Therefore, studies have investigated whether mitochondrial Ca^2+^ is involved in the release of pro-apoptotic proteins. The ceramide treatment of HeLa cells promoted Ca^2+^ release from the endoplasmic reticulum and loading into mitochondria, resulting in swelling and fragmentation of organelles and cytochrome C release. When bcl-2 is overexpressed and endoplasmic reticulum calcium levels are reduced, cytochrome C release is prevented [79]. Additionally, Ip3-mediated physiological Ca^2+^ signals are converted by ceramide into apoptosis-inducing factors [80]. Type 3 IP3Rs (IP3R-3) is located at MAM and induces apoptosis by preferentially transmitting apoptotic Ca^2+^ signals to mitochondria. Apoptosis was blocked by silencing IP3R-3 expression, and Ip3R-3 downregulation significantly reduced agonist-induced mitochondrial Ca^2+^ uptake [81,82].

mPTP is a multiprotein complex consisting of the VDAC located in the outer membrane of mitochondria, ANT located in the inner membrane of mitochondria, and matrix protein cyclophilin D (CypD). VDAC and ANT form contact sites on the outer mitochondrial membrane (OMM) and IMM. Other proteins, including hexokinase [83], the mitochondrial benzodiazepine receptor [84], Bax [85], and CK [86] are typically associated with and regulate IMM and OMM. ANT is considered to be the key to opening mitochondrial permeability transition pore [87]. However, it has been found that ANT deficiency did not block Ca^2+^-induced permeability transition [88]. VDAC was also considered to be dispensable in Ca^2+^-induced MPT and mitochondrial-dependent cell necrosis [89]. In contrast, the downregulation of cyclophilin D was found to be critical for MPT-mediated cell necrosis [90]. CypD is the most well characterized regulator of mPTP. mPTP inhibition by targeting CypD protects mouse cells against the death response to specific diseases [90,91,92,93]. Growing evidence shows that CypD is a regulator of mitochondrial Ca^2+^ and participates in the regulation of mitochondrial Ca^2+^ homeostasis through low conductance PTP opening. CypD initiates mitochondrial depolarization by activating low-conductivity PTP, generating Ca^2+^ waves that release Ca^2+^ from one mitochondrion to another [94]. CypD-deficient mice have been shown to have higher matrix Ca^2+^ levels, which may be related to the decreased opening of mPTP [95]. Cyclosporine A inhibits PTP opening by binding to matrix CypD, thus preventing PTP from binding to ANT. Studies have shown that mPTP is a node of cell death, and functions by integrating the energy metabolism and cell-death mechanism.

## 4. Mitochondrial Ca^2+^ Homeostasis and Mitochondrial ROS Emission

ROS are defined as molecules or ions formed by the incomplete one-electron reduction of oxygen. Free radicals, such as superoxide, hydroxyl radical, and singlet oxygen, and non-radical species, such as hydrogen peroxide, are ROS. Mitochondria are the main region of ROS generation. Oxygen free radicals are highly reactive and can damage cellular components such as proteins, lipids, and nucleic acids. During electron transport, electrons may leak from the reducing element of the respiratory chain and react with oxygen to form ROS. ROS play an important role in cell-signal transmission [96] but more in the generation of oxidative stress [96]. The imbalance between ROS production and ROS detoxification causes mitochondrial oxidative stress. Regulating ROS production is beneficial to signal transduction and other physiological functions. However, if ROS production is not regulated, they can cause oxidative stress, cell damage, and ultimately cell death. The ETC complexes I and III produce superoxide anion radicals during respiration, which are then decomposed into hydrogen peroxide (H_2_O_2_) by Mn^2+^-dependent superoxide dismutase [97,98,99,100]. Glutathione peroxidase (GPX) and thioredoxin/peroxiredoxin systems detoxify H_2_O_2_ using reduced NADPH (from NADP-dependent isocitrate dehydrogenase and nicotinamide nucleotide transhydrogenase). Isocitrate dehydrogenase and nicotinamide nucleotide transhydrogenase control the regeneration of NADPH in the TCA cycle [101,102]. Glutathione redox conjugate (GSH/GSSG) is the main redox buffer; GSH is a cysteine-containing tripeptide that can directly scavenge ROS or act as a cofactor of glutathione peroxidase. Glutathione peroxidase oxidizes glutathione to reduce H_2_O_2_. Additionally, Ca^2+^ has been reported to activate catalase and GSH reductase, interact with calmodulin (CaM), and then interact with enzymes involved in ROS homeostasis or the release of GSH in the early stage of PTP opening [21,103]. Furthermore, Ca^2+^ stimulates the TCA cycle and oxidative phosphorylation by increasing respiration rate, thereby enhancing ROS production at the respiratory chain complex [104]. It has been suggested that Ca^2+^ may also indirectly lead to ROS production and can activate nitric oxide synthase to produce NO and inhibit complex IV [105]. In addition, Ca^2+^ activated PTP may inhibit complex III due to the dislocation and loss of cytochrome C. Both Ca^2+^ and cytochrome c compete for cardiolipin binding sites, disrupting electron transfer and increasing ROS production [106]. Therefore, Ca^2+^ enhances ROS production by increasing respiratory rate and reducing substrate concentration. Moreover, Ca^2+^ also activates VDAC [107]. Therefore, mitochondrial Ca^2+^ can induce the TCA cycle, balance energy supply and demand, and can enhance oxidative stress. It has been reported that ROS production in isolated mitochondria increases after PTP activation, despite the requisite mitochondrial uncoupling [108]. The opening of PTP (triggered by Ca^2+^) is believed to cause conformational changes in complex I such that when electrons are provided to complex I, H_2_O_2_ formation increases, and the passage of electrons through complex I may be inhibited [109]. Another important role of the PTP opening is to produce antioxidant capacity and prevent the release of H_2_O_2_ [25]. The physiologically stable state of cardiac mitochondria is an intermediate redox state. The intermediate redox state prevents excessive ROS generation in the ETC under high reduction conditions [110,111] and prevents the loss of antioxidant capacity under high oxidation conditions [25,112,113,114]. In addition, there are other sources of ROS production in mitochondria. A-ketoglutarate dehydrogenase complex (KGDHC) plays a special role in Ca^2+^-induced mitochondrial ROS production [115]. Furthermore, Ca^2+^ has been shown to activate ROS production through isolated KGDHC [116] and other well-known mitochondrial free calcium concentration ([Ca^2+^]m)-regulated TCA cycle enzymes (isocitrate dehydrogenase, α-ketoglutarate dehydrogenase, and pyruvate dehydrogenase) [104]. In addition, ROS may play a necessary role in regulating Ca^2+^ signaling. Just as Ca^2+^ plays a role in ROS production, cellular redox states can significantly modulate Ca^2+^ signaling [114]. ROS can oxidize and regulate ryanodine receptors (RyR), inositol 1,4,5-triphosphate receptors (IP3R) channels, SERCA, plasma membrane Ca^2+^-ATPase (PMCA), NCX, and other Ca^2+^ transporters [114]. Therefore, the mechanism of the mitochondrial Ca^2+^ regulation of mitochondrial ROS production is important, and the bidirectional regulation mechanism between them must be studied further.

## 5. Mitochondrial Ca^2+^ Signaling and Autophagy

An increasing number of studies have shown that mitochondrial Ca^2+^ signaling plays a fundamental role in autophagy regulation [117,118,119]. Recently, mitochondrial Ca^2+^ has been identified as a potential specific signal regulating mitophagy [120]. It has been reported that the downregulation of ER to mitochondrial Ca^2+^ transfer can effectively decrease Parkin-mediated mitophagy [121]. Several studies have elucidated the relationship between mitochondrial Ca^2+^ signaling and autophagy/mitophagy through the cell model of mitochondrial diseases [122]. The dysregulation of Ca^2+^ in MAMs leads to abnormal autophagy [123]. The disruption of Ca^2+^ signaling between the ER and mitochondria can interfere with cell bioenergy and induce autophagy [120]. Studies have shown that *mul1* loss leads to ER-MITO decoupling, resulting in Ca^2+^ homeostasis imbalance, mitochondrial fragmentation, and mitophagy [124]. MAM is a platform that facilitates the formation of autophagy. Studies have shown that IP3Rs can transfer Ca^2+^ to mitochondria, activating the core metabolic pathways, as well as increasing the sensitivity of apoptosis and inhibiting basic autophagy [125]. The IP3-induced Ca^2+^ release enhances autophagy flux by providing cytoplasmic Ca^2+^ for autophagy in response to a variety of cellular stresses, including nutritional starvation, the rapamycin inhibition of chemomechanical targets, or drug therapy [125]. The interruption of Ca^2+^ transport from ER to mitochondria causes adenosine 5′-monophosphate (AMP)-activated protein kinase (AMPK) translocation to MAM and the activation of autophagy through Beclin-1 (BECN) [123]. Some studies have explored the correlation between mitochondrial Ca^2+^ uptake and autophagy. Muscle-restricted silencing of MCU inhibited mitochondrial Ca^2+^ and partly inhibited autophagy flux. In addition, the deletion of Atg7, an essential autophagy gene in the skeletal muscle, leads to the accumulation of dysfunctional mitochondria and greatly reduces the accumulation of mitochondrial Ca^2+^. Thus, reduced MCU activity blocks autophagy flux, and the loss of autophagy further damages mitochondrial Ca^2+^ signaling, leading to a vicious cycle [126]. Furthermore, MCU-regulator 1 (MCUR1) is a key component of the mitochondrial Ca^2+^ transport channel complex responsible for mitochondrial Ca^2+^ uptake. Loss in MCUR1 has been shown to disrupt phosphorylation, reduce intracellular ATP, and activate AMP kinase-dependent pro-survival autophagy [127]. Previous studies have shown that ITPR-mediated Ca^2+^ transport disruption stimulates autophagy [128]. The overexpression of vesicle-associated membrane protein-associated protein B (VAPB) or microtubule dynamics regulator 3 (RMDN3) enhances ER and mitochondrial contact. Additionally, VAPB-RMDN3 regulates autophagy by promoting Ca^2+^ exchange between the ER and mitochondria. The inhibition of Ca^2+^ exchange between ER and mitochondria by ITPR antagonists or siRNA-targeting MCU can eliminate the effects of VAPB and RMDN3 overexpression on autophagy [129]. Therefore, it is increasingly evident that mitochondria play a fundamental role in Ca^2+^ homeostasis and autophagy regulation in cells.

## 6. Mitochondrial Ca^2+^ Uniporter (mtCU) Protein Complex and Mitochondrial Ca^2+^ Homeostasis

High cytoplasmic Ca^2+^ microdomains is a prerequisite for mitochondrial Ca^2+^ uptake [130], and some researchers have found that Ca^2+^ uptake occurs due to the activation of pathways that may not lead to local increases in the cytoplasmic Ca^2+^ concentration [131]. Although the physiological and pathological significance of Ca^2+^ signaling pathways have been debated, recent studies have found that mitochondrial Ca^2+^ uptake and release mechanisms are central to cellular Ca^2+^ homeostasis [132,133]. VDAC and mitochondrial calcium monoporter (MCU) are two main channels that mediate Ca^2+^ influx into mitochondria [40,41,134]. VDAC is a mitochondrial outer-membrane protein responsible for the transport of Ca^2+^ to the intermembrane space. Subtypes of VDAC include VDAC1, VDAC2, and VDAC3. Of these, VDAC1 has been studied most, while information on VDAC2 and VDAC3 is limited [52], and VDAC1 has high Ca^2+^ permeability, which permits Ca^2+^ to enter and leave the mitochondria, affecting various processes of the cell [135]. Furthermore, VDAC1 plays an important role at the junction of mitochondria and endoplasmic reticulum, promoting the entry of Ca^2+^ from ER into mitochondria and regulating the death pathway of apoptotic cells. As early as the 1960s, mitochondria were identified to be organelles with the ability to accumulate Ca^2+^ [136,137]. Vasington and Murphy first demonstrated the ability of mitochondria to accumulate Ca^2+^ in the early 1960s and speculated that mitochondrial Ca^2+^ accumulation depended on respiration and phosphorylation [137]. Over the past decade, there have been several major discoveries in the understanding of the components of Ca^2+^ transport systems. In 2009, Ca^2+^/H^+^ exchangers (letm1) were first discovered [138]. A year later, mitochondrial Ca^2+^ uptake 1 protein (MICU1) [139], which regulates the entry of Ca^2+^ into mitochondria, and NCX [140], which mediates the release of Ca^2+^ from mitochondria, were discovered. In 2011, two research groups found that the Ca^2+^ channel protein subunit MCU, responsible for Ca^2+^ entry into mitochondria, is sensitive to ruthenium red [40,41]. In the following two years, more regulators of Ca^2+^ entry into mitochondria, including mitochondrial calcium uniporter dominant negative β (MCUb), MICU2, MCUR1, essential MCU regulator (EMRE), and Solute carriers—solute carrier 25A23 (SLC25A23) (Mg^2+^/ATPPi Porter), were discovered [129,141,142,143,144]. These findings suggest that the uptake of Ca^2+^ by mitochondria is mediated by a macromolecular structure, now known as the mitochondrial Ca^2+^ uniporter (mtCU), which can be inhibited by lanthanide or ruthenium red [65]. In mammals, the uniporter complex is composed of four core components—pore-forming MCU, gatekeeper MICU1, and MICU2, and auxiliary EMRE subunits necessary for calcium transport [145]. Recently, the cryo-EM structure of human mtCU holocomplex in low/high-Ca^2+^ conditions was reported [146,147]. The stoichiometric ratios of MCU, EMRE, MICU1, and MICU2 determined at low Ca^2+^ concentration were 4:4:1:1. A Ca^2+^-conducting hole is formed by the tetramerization of MCU, and EMREs are attached to the periphery of the hole around a central approximate quadruple symmetry axis. Both MICU1 and MCU form an extensive interaction surface to close the entrance of the inter membrane space of the hole, while MICU2 combines with MICU1 from the side without contacting MCU [145]. The Ca^2+^ in mitochondria can also be released through the Na^+^/Ca^2+^ or Ca^2+^/H^+^ exchanger [148]. The uptake of Ca^2+^ is driven by the mitochondrial membrane potential, and is electrically neutral in the release of proton exchange or sodium [149]. When intracellular Ca^2+^ concentration increases, mitochondria can accumulate considerably larger amounts of Ca^2+^ under pathological conditions. It has been found that the mitochondrial Ca^2+^ flow regulates the spontaneous electrical activity of ventricular myocytes [150]. Additionally, Ca^2+^ plays a key role in the excitation–contraction coupling of the myocardium [28] and flows into the cytoplasm from the extracellular space through voltage-gated L-type Ca^2+^ channels, triggering the opening of RyR2 on the SR toward the t-tubules, promoting the release of Ca^2+^ in the SR (i.e., Ca^2+^ induced Ca^2+^ release), thus causing the transient increase in the intracellular calcium concentration, which is called “calcium transient”. Transient calcium ions promote the binding of Ca^2+^ to troponin C, which triggers cardiac contraction. Elevated cytoplasmic Ca^2+^ enter the SR cavity through the SERCA or are pumped out of the cell via NCX [132]. Increased Ca^2+^ cycling is associated with increased ATP consumption. Increasing evidence suggests that the transient increase in [Ca^2+^]m of myocardial mitochondrial Ca^2+^ uptake on the mitochondrial matrix acts as a regulatory signal to ensure the balance of energy supply and demand (i.e., excitation metabolism coupling). However, the importance of mitochondrial Ca^2+^ buffering capacity, the kinetics of mitochondrial Ca^2+^ uptake/release, and the molecular mechanism of [Ca^2+^]m-mediated ATP and ROS production remain controversial.

Previous studies have revealed that cardiomyocytes promote mitochondrial Ca^2+^ influx through mitochondrial Ca^2+^ channels and transporters [132,151]. In the heart, the main component of Ca^2+^ influx is the MCU complex, and the main complex mediating mitochondrial Ca^2+^ efflux is the mitochondrial NCX. However, it is generally accepted that myocardial cells primarily uptake Ca^2+^ through MCU complexes [152]. The MCU complex is an important regulator of [Ca^2+^]m and plays an important role in regulating mitochondrial Ca^2+^ homeostasis [153]. The MCU gene was first discovered and reported in 2011 [41]. The highly conserved MCU (CCDC109a) gene encodes the 40 kDa mtCU, which forms a Ca^2+^ channel in mitochondria and exists in almost all eukaryotes except for some protozoa and fungi [154]. MCU consists of two helical domains (CC) and two transmembrane domains connected by a short loop (9 amino acid residues) containing a highly conserved dimer motif [40,41]. The N-terminal and C-terminal of the protein extend to the mitochondrial matrix. The loop of the transmembrane domain extends into the mitochondrial intermembrane space and is responsible for Ca^2+^ transport (Glu257, Asp260, and Glu264) and ruthenium red binding (Ser259) [41,133,141]. The mutation of these residues affects the transport capacity of Ca^2+^ and reduces sensitivity to ruthenium red. Studies have revealed that changes in MCU expression or activity in non-cardiomyocytes have no effect on the mitochondrial membrane potential, oxygen consumption, ATP production, and mitochondrial morphology [155]. Subsequent studies revealed that the MCU complex was primarily responsible for mitochondrial Ca^2+^ influx, and MCU knockdown reduced mitochondrial Ca^2+^ uptake, while MCU overexpression restored mitochondrial Ca^2+^ uptake in knockdown cells [156,157,158]. Unlike MCU, MCUb has been reported to be an endogenous negative regulatory subunit of the MCU complex [141]. MCUb forms a heterooligomer with MCU, and the binding of MCUb to MCU reduces the Ca^2+^ permeability of the MCU complex [141,143]. Moreover, the overexpression or knockdown of MCUb and subsequent changes in the ratio of MCUb:MCU lead to a significant decrease or increase, respectively, in Ca^2+^ in mitochondria [159]. In addition to MCUb, some auxiliary subunits of MCU complexes have also been discovered, including mitochondrial Ca^2+^ uptake 1 protein (MICU1) [139], MICU2 [142], MICU3 [142], EMRE [143], and MCU- regulator 1 (MCUR1) [127]. Recent studies have revealed that MCUb can replace MCU to regulate the stoichiometry of mtCU and has important effects on mitochondrial Ca^2+^ uptake and cellular physiology [160]. The overexpression of MCUb can reduce the infarct size caused by IR injury [160]. Furthermore, MICU1 is the first auxiliary subunit of the MCU complex. Previous studies have revealed that MICU1 has a regulatory function and acts as a gatekeeper in the MCU complex. Additionally, MICU1 keeps the MCU pore closed under normal conditions [160], while MICU1 knockdown increases the basal mitochondrial Ca^2+^ level [161], and even in the case of low cytoplasmic Ca^2+^, MICU1 silencing leads to constitutive mitochondrial Ca^2+^ overload [127,162]. However, some studies have revealed that MICU1 knockdown inhibits mitochondrial Ca^2+^ uptake, suggesting that MICU1 plays a positive regulatory role in the MCU pore [139,163]. Similar to MICU1, MICU2 is also considered a gatekeeper. Whether MICU1 or MICU2 plays the predominant role in gatekeeping is still controversial [155]. In the heart, MICU1 expression is relatively low, whereas MICU2 expression in the heart is higher than that in other organs, suggesting that MICU2 plays a more important role than MICU1 in the physiological and pathological conditions of the heart [132]. Furthermore, MICU2 deletion was found to prolong Ca^2+^ removal and the diastolic time of cardiomyocytes, presenting with mild diastolic dysfunction in vivo [164]. Another important auxiliary subunit in the MCU complex is EMRE, which is considered as a bridge between MCU and MICU1/2. It transmits cytosolic free calcium concentration ([Ca^2+^]c) changes to the MCU complex and activates mitochondrial in situ Ca^2+^ uptake [143,165]. EMRE has been proposed to be a [Ca^2+^] sensor on the matrix side [166]. Furthermore, MCUR1 is a regulatory protein of the MCU complex. Some studies have revealed that MCUR1 is the scaffold protein of the MCU complex, which is essential for cell bioenergy and function [127,167]. Shoubridge et al. showed that MCUR1 knockout inhibits the activity of the ETC by reducing the assembly and activity of cytochrome C oxidase (complex IV), suggesting that the effect of MCUR1 gene knockout on MCU activity may be indirectly affected by changes in the mitochondrial membrane potential [168,169]. In summary, the MCU complex is the main means of mitochondrial Ca^2+^ uptake in various cells/tissues, including adult ventricular myocytes/hearts. Induced cardiomyocytes isolated from MCU knockout mice were found to still uptake Ca^2+^. The expression of MCU was reduced to 20%, and mitochondrial Ca^2+^ uptake was almost completely lost (10–20% of adult ventricular myocytes in the control group). Moreover, the further treatment of MCU knockout cardiomyocytes with Ru360, which is an inhibitor of MCU, did not further inhibit the residual mitochondrial Ca^2+^ uptake, suggesting that MCU is not the only mechanism that mediates mitochondrial Ca^2+^ uptake in cardiomyocytes [157]. The MCU has recently been shown to be a redox sensor whose activity increases following oxidation. Furthermore, Cys-97 plays a unique role in ROS sensing and MCU activity regulation. The oxidation of MCU-Cys-97 promotes the formation of its oligomer, resulting in sustained MCU channel activity, enhanced [Ca^2+^]m uptake rate, increased mtROS, and enhanced cell death induced by [Ca^2+^]m overload [170]. In heart disease, the decrease in Ca^2+^ transient amplitude and the increase in cytoplasmic Na^+^ play an important role in decreasing mitochondrial Ca^2+^ [48]. The increase in mitochondrial Ca^2+^ observed with isolated mitochondria may be due to the increase in MCU activity due to its post-translational modification. It has been shown that α-adrenergic stimulation of ROS/Ca^2+^-dependent proline-rich tyrosine kinase Pyk2 translocates from the cytosol to mitochondria, promoting the formation of a tetramer MCU pore and accelerating the uptake of Ca^2+^ by mitochondria [159]. Therefore, other mechanisms of mitochondrial Ca^2+^ influx need to be further studied.

## 7. Other Mitochondrial Ca^2+^ Influx Mechanisms on Mitochondrial Ca^2+^ Homeostasis

Before the discovery of the MCU complex, several different types of mitochondrial Ca^2+^ uptake mechanisms were investigated using various experimental methods [171]. Skeletal muscle type RyR type 1 (RyR1) is expressed in the mitochondrial membrane and plays a role in Ca^2+^ uptake in cardiomyocytes [172]. Investigations of the regulatory effect of mitochondrial RyR1 on mitochondrial morphology/function of cardiomyocytes revealed that transient or stable RyR1 overexpression was partially localized in mitochondria. In addition, the overexpression of RyR1 instead of MCU or RyR2 led to mitochondrial fragmentation [173]. These results suggest that RyR1 possesses mitochondrial localization signals that regulate mitochondrial morphology and Ca^2+^-induced ATP production in cardiac H9c2 myoblasts [173]. Other mechanisms of mitochondrial Ca^2+^ uptake include rapid uptake mode (RAM) [174,175], Ca^2+^-selective conductivity (mCa1 and mCa2) [176,177,178], leucine zipper EF-hand containing transmembrane protein 1 (letm1) [125] and coenzyme Q10 [179]. Additionally, mCa2, RAM, and RyR1 in cardiomyocyte mitochondria may mediate mitochondrial Ca^2+^ uptake through MCU-independent pathways possibly involving different molecular mechanisms [132].

Furthermore, Ca^2+^ is also thought to be transported into mitochondria by proteins other than Ca^2+^ uniporter, including uncoupling proteins 2 and 3 (UCP2 and UCP3) [180]. Uncoupling proteins (UCPs), embedded in the inner membrane of mitochondria, belong to the mitochondrial ion transport superfamily [181]. Accumulating evidence suggests that UCP2 and UCP3 play roles in many cellular processes, including mitochondrial free-radical production, apoptosis, the regulation of hormone secretion, and glucose and fatty acid metabolism. Due to the processes of overexpression, knockdown and mutation of UCP2 and UCP3, it has been found that they are elementary for mitochondrial Ca^2+^ sequestration and are essential for mitochondrial Ca^2+^ uptake [182]. It has also been suggested that UCP2/3 expression levels are critical to the ability of mitochondria to sequester entering Ca^2+^ [183]. Studies have shown that UCP3 acts as a complex molecular switch with different sensitivities to high and low levels of Ca^2+^. Its contribution to mitochondrial Ca^2+^ uptake depends on the intermembrane loop 2 (IML2) [184]. A subsequent article by Waldeck-Weiermair et al. provided further insight into the role of UCP2 and UCP3 in mitochondrial Ca^2+^ homeostasis. They found that the down-regulation of UCP2 and UCP3 only reduced the mitochondrial Ca^2+^ uptake of intracellularly released Ca^2+^ in response to histamine, a mobilization agonist of inositol-1,4,5-triphosphate (IP3). Therefore, the significant contribution of UCP2 and UCP3 to mitochondrial Ca^2+^ uptake depends on the source and pathway of the increase in intracellular Ca^2+^ [183,184]. Subsequent electrophysiological analyses have shown that UCP2 and UCP3 regulate MCU-dependent Ca^2+^ currents in mitoplasts [185], and these findings are further supported by other groups, these studies suggest that UCP2 is involved in the mitochondrial Ca^2+^ uptake current in mitoplasts of mouse cardiomyocytes [176,178]. However, subsequent studies have shown that UCP3 is not a mitochondrial Ca^2+^ uniporter, and instead negatively regulates SERCA activity by limiting mitochondrial ATP production [186]. UCP2 is thought to play a neuroprotective role by stimulating mitochondrial biogenesis and preventing cell death by reducing membrane potential and calcium influx into mitochondria [187]. Phenotypes of UCP2 knockout mice showed increased susceptibility to Ca^2+^-mediated ventricular arrhythmias, suggesting that UCP2 plays an important role in cardiac electrophysiology [188].

## 8. MAMs and Mitochondrial Ca^2+^ Homeostasis

**Figure 3:**
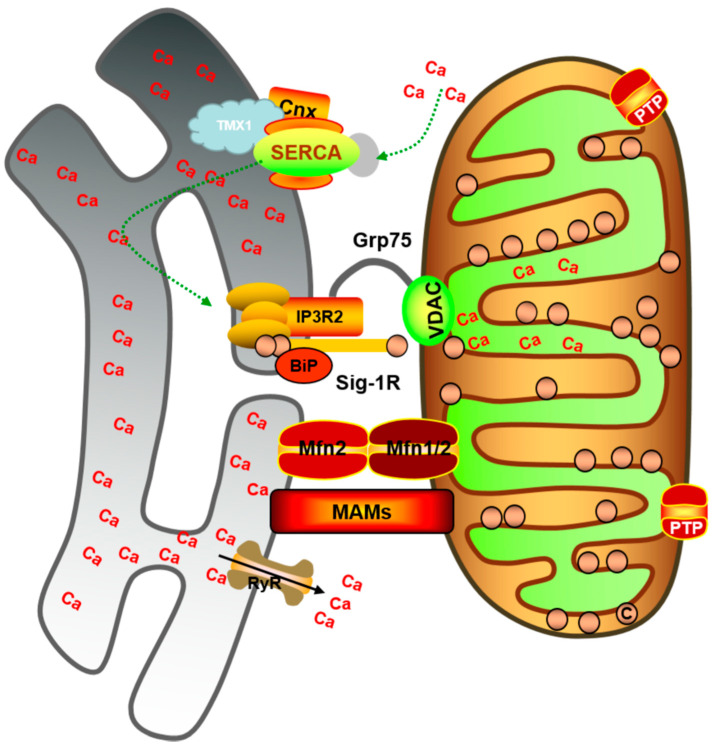
Mitochondria-associated membrane (MAM) protein complex regulating Ca^2+^ in cardiomyocytes. There are many MAM complexes between the endoplasmic reticulum (ER) and mitochondria. The IP3R2-Grp75-VDAC complex is an important MAM complex. The Grp75 acts as a bridge between IP3R2 on ER and voltage-dependent anion channel 1 (VDAC1) on the mitochondrial outer membrane to regulate mitochondrial Ca^2+^ uptake. ER protein sigma-1 receptor (Sig-1R) is a Ca^2+^ sensitive and ligand-operated receptor chaperone located in MAM. The Sig-1Rs form a complex with another molecular chaperone BiP on MAM. The Ca^2+^ in ER is depleted or stimulated by ligands, Sig-1R is separated from BiP, producing a prolonged Ca^2+^ signal into mitochondria-dependent on IP3Rs. The increased expression of Sig-1Rs can counteract the ER stress response, while the decrease in Sig-1Rs can enhance apoptosis. Mitochondrial GTPase mitofusin (MFN2) is enriched in the MAM and is also located in the ER, while MFN2 is an endoplasmic reticulum-mitochondria tether. In the ER, it interacts with mitofusins on mitochondria to form an interorganellar bridge. Sarco/endoplasmic reticulum Ca^2+^-ATPase (SERCA) 2b is located in the ER and is responsible for moving Ca^2+^ from the cytoplasm into the ER. Calnexin (CNX) is a quality control partner of the ER, which interacts with SERCA2b. ER-localized thioredoxin-related transmembrane protein 1 (TMX1) interacts with SERCA2b in a thiol-dependent manner to reduce SERCA activity under oxidative conditions. Ryanodine receptor type 2 (RyR2) channel of the sarcoplasmic reticulum is a Ca^2+^ outflow channel of ER. Its activation results in the release of a large amount of Ca^2+^ by the sarcoplasmic reticulum and the transient increase of cytoplasmic Ca^2+^.

ER is the main storage site of Ca^2+^ in cells [189]. ER and mitochondria play an important role in the transmission of Ca^2+^ signals in physiological and pathological processes [190]. In the 1950s, early signs of a link between the ER and mitochondria were described [191]. In recent years, a physical coupling between mitochondria and ER, called MAM, has been discovered [192]. The number, length, and thickness of the ER in contact with mitochondria are important parameters in determining MAM function [193]. MAM dysfunction is associated with disturbances in calcium homeostasis, phospholipid metabolism, mitochondrial functions, and dynamics [193]. The importance of MAM in Ca^2+^ homeostasis has been established [194]. MAM is a specific microdomain of Ca^2+^ transfer. Cardiomyocyte mitochondria are closely related to the SR. MAM is a dynamic structure that promoted efficiency in the Ca^2+^ transfer from ER to mitochondria. Several proteins are involved in Ca^2+^ transmission [195]. MAM is a complex formed by the precise regulation of Ca^2+^ exchange between ER and mitochondria through the recruitment of different mitochondria-related proteins and plays an important role in maintaining mitochondrial Ca^2+^ homeostasis and ultimately regulating the function and survival of cells [196] (Figure 3). The proteins involved in Ca^2+^ transport in MAM include the ER Ca^2+^ releasing protein, mitochondrial outer membrane-associated protein, and mitochondrial inner membrane-associated protein [197]. Furthermore, Ca^2+^ efflux from the ER reaches the mitochondrial matrix through VDAC channels on the OMM and accumulates in the mitochondrial matrix through MCU complexes. The MAM chaperone glucose-regulated protein 75 (GRP75) links the Ca^2+^ efflux of ER with VDAC1 on the OMM to regulate mitochondrial Ca^2+^ uptake [198]. Therefore, GRP75 is the bridge connecting IP3R and VDAC1 [199]. Studies have revealed that when the expression of GRP75 in cells is reduced, the functional coupling between ER and mitochondria is eliminated, and the uptake of Ca^2+^ is affected. This indicates that GRP75 plays an important role in Ca^2+^ communication between ER and mitochondria [200]. An important element is IP3R, which is an inositol triphosphate-dependent Ca^2+^ channel located on the ER membrane that controls the outflow of Ca^2+^ from ER into the cytoplasm. It forms the IP3R-GRP75-VDAC1 complex with IP3R and VDAC1 [201]. VDAC is a mitochondrial outer membrane protein, which together with MCU, regulates Ca^2+^ influx into mitochondria. The protein complex is responsible for Ca^2+^ transfer from ER to mitochondria [200,201]. It has been reported that Ca^2+^ transport to mitochondria requires an MCU known as the IP3R-GRP75-VDAC-MCU Ca^2+^ regulatory axis [194,201]. Because mitochondrial Ca^2+^ is a key regulator involved in many biological functions, the IP3R-GRP75-VDAC-MCU complex may play an important regulatory role in various cellular functions. It has recently been shown that proteins such as transglutaminase type 2 (TG2) [199], CypD, and DJ-1 [202] interact with the IP3R-GRP75-VDAC1 complex to regulate Ca^2+^ transfer from ER to mitochondria. Studies have revealed that CypD, as a member of the ER and mitochondrial contact site VDAC1-GRP75-IP3R1 complex, promotes Ca^2+^ transfer in the two organelles, inhibits CypD, IP3R, and GRP75, reduces protein interactions in the complex, and slows down mitochondrial Ca^2+^ overload. It is suggested that mitochondrial Ca^2+^ uptake plays an important role in cardiac ischemia-reperfusion injury and can be used as a target for cardiac protection [203]. Phosphofurin acidic cluster sorting 2 protein (PACS-2) is a multifunctional cytoplasmic protein that induces apoptosis [204,205]. However, whether PACS-2 can directly attach to MAM is not clear. The loss of PACS-2 has been reported to reduced ER-mitochondrial contact and mitochondrial fragmentation [204]. Sigma non-opioid intracellular receptor 1 (SigR1) and tespa1 are also important proteins that bind the IP3R-GRP75-VDAC-MCU calcium channel on MAM. Furthermore, SigR1 overexpression increases Ca^2+^ efflux from ER by interacting with ankyrin and the ER chaperone protein BiP [206,207]. Tespa1 regulates Ca^2+^ levels by binding to IP3R and GRP75. The knockdown of Tespa1 reduces mitochondrial and cytoplasmic Ca^2+^ levels [208]. The Fun14 domain containing 1 (FUNDC1) is another protein that regulates the dynamics of MAM [209]. While IP3R2 binds FUNDC1 to regulate SR Ca^2+^ release [210], FUNDC1 competitively binds DRP1 during early hypoxia. In the late stage of hypoxia, FUNDC1 separates from calnexin and binds DRP1, leading to mitochondrial fission and mitophagy [211]. Presenilin (PS) is a multifunctional protein whose mutation leads to familial Alzheimer’s disease [212]. PS interacts with MFN2 to regulate MAM under Ca^2+^ overload. The PS2 gene mutation affects mitochondrial Ca^2+^ delivery [213,214]. In conclusion, MAM plays an important role in mitochondrial Ca^2+^ overload and cell necrosis.

## 9. Molecular Mechanism Underlying Mitochondrial Calcium Efflux Regulation

The mitochondrial Ca^2+^ efflux mechanism involves the NCX and H^+^/Ca^2+^ exchangers. The kinetic characteristics of mitochondrial Ca^2+^ uptake in different tissues are very similar, but mitochondrial calcium efflux mechanism in these tissues is different, mainly by H^+^/Ca^2+^ exchange in non-excitable tissues such as liver and kidney, and mainly by Na^+^/Ca^2+^ exchange in excitable cells such as neurons and striated muscles [215,216]. The molecular nature of 2H^+^/Ca^2+^ exchangers remains controversial, but studies have suggested that Letm1 may be a plausible candidate [217]. A previous study showed that silencing *Letm1* disrupts Ca^2+^/H^+^ exchange in *Drosophila S2* and HeLa cells [138]. On the other hand, mitochondrial Ca^2+^ dynamics are also affected by Letm1 [180]. Letm1 moves these two cations across the membrane in a 1 Ca^2+^/2 H^+^ ratio and is therefore a Ca^2+^/H^+^ antiporter [217]. Some studies have shown the mitochondrial localization of NCX, known as NCLX. NCLX is a 100-kDa dimer protein expressed on the inner membrane of brain and heart mitochondria. Its knockdown or overexpression has been shown to significantly reduce or increase mitochondrial Ca^2+^ efflux [218]. Lungo et al. found that inducible cardiac-specific knockout of NCLX decreases the mitochondrial Ca^2+^ efflux rate, mitochondrial Ca^2+^ overload, increased cell necrosis, and sudden death of the animal, indicating that NCLX is key to the regulation of mitochondrial Ca^2+^ [219]. This is consistent with previous studies suggesting that Ca^2+^ efflux from cardiac mitochondria is dependent on cytosolic Na^+^. NCLX knockout has been found to cause sudden death in mice owing to myocardial dysfunction and heart failure. The cardiac pathologic causes are superoxide production and necrotic cell death caused by mitochondrial Ca^2+^ overload and can be prevented by the inhibition of the activation of mPTP [56]. The overexpression of NCLX in mouse hearts by transgenic methods can effectively remove mitochondrial Ca^2+^, prevent permeability transition, and protect against necrosis and heart failure caused by myocardial ischemia [219].

The opening of mPTP has been shown to promote the balance of cofactors and ions, including Ca^2+^, in the IMM and cause the destruction of mitochondrial membrane potential and ATP production, as well as mitochondrial swelling until the breaking of the OMM [190]. Therefore, mPTP is the main cause of reperfusion injury and an effective target of cardiac protection [220]. mPTP is considered to be involved in Ca^2+^ efflux under physiological and pathological conditions [221]. In adult rat ventricular myocytes, mPTP allows for the dissipation of Ca^2+^ through mitochondrial membrane potential [222]. In addition, cyclophilin D is a known regulator of mPTP. mPTP has been shown to act as a Ca^2+^ valve to limit an increase in myocardial mitochondrial Ca^2+^ in cyclophilin D-deficient mice [95]. In the process of the physiological excitation–contraction coupling of cardiac myocytes, cyclophilin D can stimulate the transient opening of the mPTP pore, promoting Ca^2+^ efflux in addition to the NCLX regulation of metabolism in individual mitochondria [223]. The opening of mPTP promotes Ca^2+^ exchange between mitochondria and matrix, coupled with proton counterflow into matrix space [224]. These studies suggest that mPTP may be important in mitochondrial Ca^2+^ efflux under cardiac physiological and pathological conditions.

## 10. Mitochondrial Ca^2+^ Dyshomeostasis and Cardiac Pathological Remodeling

Mitochondrial Ca^2+^ plays a dual role in regulating cardiac function. Cardiac mitochondrial Ca^2+^ deficiency can impair mitochondrial function, reduce energy supply, and lead to cell damage or death [225]. In addition to energy supply, mitochondrial Ca^2+^ homeostasis plays an important role in other biological functions of cardiomyocytes. For example, mitochondria can buffer intracellular Ca^2+^ to stimulate cardiac excitation–contraction coupling [226,227]. When this function of mitochondria is damaged, the contractile force of cardiomyocytes is disturbed, leading to cardiac insufficiency [226]. Pathological stress such as ischemia, infarction, and pressure overload can induce excessive Ca^2+^ accumulation in cardiomyocytes and lead to mitochondrial Ca^2+^ overload [228]. The maintenance of mitochondrial Ca^2+^ homeostasis is essential for the survival and function of cardiomyocytes.

The effect of mitochondrial Ca^2+^ on chronic heart failure has been widely studied. Additionally, Ca^2+^ treatment disorders are closely associated with heart failure (HF) [229]. Mitochondrial Ca^2+^ homeostasis disorder is a sign of heart failure [230]. In failing hearts, the impaired reuptake of Ca^2+^ by SR and increased Ca^2+^ leakage through RyR has been shown to result in a decreased amount of intracellular Ca^2+^ transients during excitation, but increased intracellular Ca^2+^ at baseline [231,232]. There is increasing evidence of intracellular Ca^2+^ overload leading to mitochondrial Ca^2+^ homeostasis damage, the opening of mPTP, increasing mitochondrial oxidative stress, the collapse of mitochondrial membrane potential, damage to ATP production, necrosis of myocardial cells, and subsequent heart failure in animal models [233]. However, the level of Ca^2+^ that leads to mitochondrial Ca^2+^ overload in heart failure and the mechanism by which it manifests heart failure remains to be determined. In addition, whether mitochondria are the main Ca^2+^ pool in the physiological and pathological process of the heart, is controversial [234]. The damage caused by mtROS has been proven to be the main pathogenic mechanism of heart failure [233]. Studies have shown that damage caused by excessive mtROS is evident in human heart-failure patients and animal models [235,236]. Mitochondrial-targeted active oxygen scavenging is beneficial for heart recovery in the heart failure animal models [236,237,238]. A series of changes in the energy metabolism and redox state occur during cardiac ischemia-reperfusion injury, including decreased ATP level, increased cytoplasmic phosphate and Ca^2+^ levels, and increased ROS release [239,240]. These conditions lead to mPTP opening. mPTP opening has been shown to be involved in tissue damage that occurs during reperfusion after ischemia by measuring mitochondrial swelling in intact hearts using radiodeoxyglucose [241]. Furthermore, the presence of antioxidants during reperfusion significantly reduced myocardial injury induced by ischemia-reperfusion, suggesting that oxidative stress is one of the causes of tissue damage underlying these conditions [242].

Some scientists have shown that mitochondrial Ca^2+^ homeostasis affects the regulatory function of PGC-1 α [243]. It is known that PGC-1 α plays a central role in the regulation of the cardiac energy metabolism by driving coupled respiration and activating mitochondrial biology because PGC-1 α is a member of a family of transcription co-activating factors [244]. Thus, mitochondrial Ca^2+^ may alter the energy metabolism and signaling within organelles by accumulating, buffering, and releasing Ca^2+^, leading to diabetic cardiomyopathy [245]. Some scientists used a type 1 STZ-induced diabetic rat model to simulate diabetic patients, and found defects in mitochondrial Ca^2+^ treatment in the model, resulting in a decreased Ca^2+^ uptake and ATP synthesis rate [246]. In addition, Tanaka et al. also confirmed a reduction in mitochondrial Ca^2+^ accumulation in animals injected with STZ [247]. It has also been suggested that the opening of cardiac mitochondrial mPTP in diabetics leads to the release of accumulated Ca^2+^ [248]. Diaz Juarez et al. reported that cardiomyocytes exposed to high glucose showed decreased mitochondrial Ca^2+^ levels and dehydrogenase activity, which may be related to low MCU levels. Low rates of mitochondrial Ca^2+^ uptake have been found in animal models of obesity and type 2 diabetes [249]. Belke et al. also found that in db/db mice, the level of Ca^2+^ and the decay rate of Ca^2+^ decreased, indicating impaired mitochondrial Ca^2+^ uptake [250]. Mitochondrial Ca^2+^ overload is believed to lead to cardiac dysfunction by promoting the production of mitochondrial ROS and the opening of mPTP, primarily via cardiomyocyte death [158,251,252]. Genetic studies have shown that ANT and the phosphate carrier (PiC) are regulators of mPTP, while F1-F0 ATP synthase is a component of the pores. These three are not only functionally coupled with mitochondrial ATP but also physically coupled with the inner membrane supercomplex called ATP synthasome [253]. The removal of any component of ATP synthase results in mitochondrial oxidative phosphorylation disorder [254,255], which affects energy production [256], leading to a series of heart diseases. ANT1 deficiency has been reported to lead to hypertrophic cardiomyopathy, myopathy, lactic acidosis, and exercise intolerance [257]. Moreover, the knockout of PiC or ANT1 in mouse hearts can lead to severe mitochondrial car-diomyopathy [254,258,259]. Human patients with PiC skeletal muscle subtype mutations have muscle weakness, lactic acidosis, hypertrophic cardiomyopathy, and a shortened lifespan [260].

Because Ca^2+^ directly inhibits glutathione reductase, the main antioxidant in the matrix, mitochondrial Ca^2+^ overload also reduces the scavenging ability of mitochondrial ROS [261]. In addition, the excessive production of ROS mediated by mitochondrial Ca^2+^ leads to the post-translational modification of Ca^2+^-handling proteins such as RyR2, and the subsequent disruption of cytoplasmic Ca^2+^ processing and mPTP opening, ultimately leading to cardiomyocyte apoptosis [56]. In myocardial ischemia/reperfusion injury, mitochondrial Ca^2+^ overload-mediated cardiomyocyte death is due to mitochondrial Ca^2+^-dependent mPTP activation [262]. Studies of an induced cardiomyocyte-specific MCU knockout model and NCLX overexpression model suggest that mitochondrial Ca^2+^ overload is the main mediator of ROS production and mPTP activation which induce ischemia/reperfusion injury and other acute heart diseases [263]. However, some studies have found that reduced mitochondrial Ca^2+^ leads to oxidative stress and cell damage under cardiac pathological conditions [264]. The intracellular sodium concentration [Na^+^]c has been shown to be increased in failing guinea pig hearts relative to [Na^+^]c in normal hearts [25,48,265]. NCLX accelerates Ca^2+^ outflow and reduces [Ca^2+^]m in failing hearts [266]. In addition, the pharmacological inhibition of NCLX restored mitochondrial Ca^2+^ treatment and cell oxidation in adult ventricular myocytes [267]. In a type 1 diabetic mouse model, MCU expression, mitochondrial Ca^2+^ uptake, and mitochondrial Ca^2+^ content in adult ventricular myocytes were significantly reduced. In addition, MCU re-expression in diabetic hearts has been found to improve the treatment and metabolism of impaired mitochondrial Ca^2+^ [268]. The study of the heart-failure model after myocardial infarction in mice showed that RyR2-mediated increased SR-Ca^2+^ leakage is accompanied by an increase in mitochondrial calcium concentration (Mito-[Ca^2+^]), suggesting that mitochondrial Ca^2+^ overload is a key determinant of heart failure [251]. Xie et al. evaluated the effect of mitochondrial Ca^2+^ influx on arrhythmia risk in non-ischemic cardiomyopathy and found that the mitochondrial Ca^2+^ level increased in ventricular myocytes of a non-ischemic heart failure model induced by hypertension in mice [269]. The level of Ca^2+^ in mitochondria isolated from an aging human heart was found to be increased, which was attributed to the post-translational modification of MCU, which leads to its function becoming impaired under the condition of oxidative stress and increased catecholaminergic tension (such as HF) [270].

## 11. Targeting Mitochondria: Mitochondrial Ca^2+^ as Drug Targets in the Treatment of Cardiovascular Diseases

The increased incidence of malignant arrhythmias can contribute to heart failure, diabetic cardiomyopathy, senile cardiac insufficiency, and hereditary diseases [271]. Abnormalities in intracellular Ca^2+^ homeostasis and mitochondrial dysfunction are considered to be key factors in the pathophysiology of these diseases [272]. Garcia Rivas et al. showed that a perfusion of the MCU inhibitor RU360 could eliminate the incidence of arrhythmias caused by ischemia/reperfusion injury in an open-chest rat model [273]. Thus, the inhibition of MCU prevents mitochondrial Ca^2+^ overload, the subsequent activation of mPTP and loss of mitochondrial membrane potential. As the MCU complex is not the only pathway for Ca^2+^ influx, an inhibition of MCU expression/function does not affect basic Mito-[Ca^2+^] or substantially affect cardiac function under basic conditions [274]. The increase in mitochondrial ROS has also been associated with arrhythmogenic effects [275].

Studies have shown that drugs targeting mitochondria interfere with mitochondrial Ca^2+^ transport and Ca^2+^-induced membrane permeability transition, inhibit the activation of the MAPK/JNK pathway, inhibit foam-cell formation, and reduce the progression of atherosclerosis [276]. CsA is an inhibitor of mPTP and can bind to CypD, a positive regulator of mPTP. The cardioprotective effect of CsA has been assessed in several reperfusion myocardial infarction model animals, but results regarding a reduction in the infarct size have been inconsistent [277]. In a Phase II trial, CsA in patients with ST-segment elevation MI (STEMI) showed promising results [278,279].

It is promising to develop therapeutic peptides targeting mitochondrial Ca^2+^ regulation. These peptides primarily include Ca^2+^ channels and pumps that regulate ER localization and small peptides or proteins that regulate mitochondrial Ca^2+^ channels, affecting Ca^2+^ transport from ER to mitochondria (Table 1). Anti-apoptotic Bcl-2 proteins also play an important role in regulating intracellular Ca^2+^ signaling. Additionally, Bcl-2 binds to the central regulatory region of IP3R through its BH4 domain, inhibiting IP3R-mediated Ca^2+^ release. The introduction of the BH4 domain of Bcl-2 (BH4-Bcl-2) as a polypeptide has been shown to inhibit IP3R-mediated Ca^2+^ release and protect cells from pro-apoptotic Ca^2+^ transfer to mitochondria [280]. An IP3R-derived peptide, Bcl-2/IP3R disruptor 2 (Bird-2), was established based on the BH4-Bcl-2 binding site in the central regulatory region of IP3R, which is located in a 20-amino acid region [280]. The mechanism of Bird-2 inducing cell death has not been elucidated. In the heart, DHPRs activates RyR by triggering Ca^2+^-induced Ca^2+^ release via Ca^2+^ influx. Further studies have shown that a short fragment of the cytosolic II–III loop of the DHPR, called peptide A, induces RyR1-mediated Ca^2+^ release [281]. The lipoamino acid conjugation of peptide A increased its cellular permeability while maintaining its structural and functional properties, making it a potential therapeutic option [282]. Some peptide toxins, called calcins, have similar effects on RyR1 gating [283]. Calcins are short cell-permeable peptides that have a high affinity for RyR1 and specifically bind and stimulate its activity. Moreover, some peptides have been found to regulate mitochondrial Ca^2+^ uptake by directly or proximally acting on VDAC and MCU. Short peptides from the N-terminal of VDAC1 and LP4 (ANTP-N-TER and ANTP-L14-15, respectively) may significantly inhibit mitochondrial Ca^2+^ uptake and lung cancer cell migration by blocking the interaction between VDAC1 and Bcl-XL, McL-1 [284], or HKI. The R-tf-d-lp4 peptide significantly increased intracellular Ca^2+^ levels, and this event was associated with VDAC1 oligomerization, cytochrome c release, and apoptotic cell death. Peptides derived from accessory protein sequences of VDAC1 show potential therapeutic applications [285]. Furthermore, BH4-Bcl-XL almost completely blocks VDAC1-mediated Ca^2+^ uptake into mitochondria, making cells more resistant to the pro-apoptotic release of Ca^2+^ from the MAM-targeted ER [286]. Owing to the effect of BH4-Bcl-XL on VDAC1 and its role as an inhibitor of RyR in ER, it can be used to treat diseases characterized by toxic mitochondrial Ca^2+^ signaling, such as ischemia-reperfusion injury [287], Alzheimer’s disease [288], and Parkinson’s disease [289]. Studies have shown that B-type natriuretic peptide (BNP) released by cardiomyocytes plays a cardioprotective role by inhibiting MCU and affecting mPTP opening [290]. The recombinant BNP peptide (Nesiritide, Natrecor) has been approved by the FDA for the treatment of acute decompensated congestive heart failure, but its clinical efficacy remains controversial. Some other peptides indirectly affect ER-mitochondrial Ca^2+^ flux or homeostasis. The fungus-derived cyclic peptide cyclosporine A (CsA) desensitizes mPTP to Ca^2+^ and inhibits pore opening. In recent years, an increasing number of CypD-selective and non-immunosuppressive derivatives of CsA (such as mtCsA, NIM811, and DEBio-025) have been developed as promising cardioprotective agents, as their ability to reduce the harmful effects of acute myocardial infarction has been observed in different models [291]. Another non-immunosuppressive analogue of cyclosporine A is Alisporivir (Ali), which inhibits MPT pore assembly by interacting with cyclosporine D [292]. Studies have shown that Ali, as a mitochondrial targeted metabolic reprogramming agent, can significantly increase Ca^2+^ retention in diabetic animals, reduce oxidative damage of heart tissue, and improve the glucose utilization rate [293]. More recently, the octapeptide RRNYRRNY (RNY) has been identified as a potential cardiac protective agent that inhibits the connexin 43 (Cx43) hemichannels in mitochondria [294]. Furthermore, Cx43 is a connexin that forms mitochondrial Ca^2+^ permeable hemichannels, contributing to a mitochondrial Ca^2+^ overload and loss of energy and ion gradients, leading to cell death [295,296]. RNY can offset the harmful effects of mitochondrial Cx43-HCs through its channel-inhibitory activity, which reduces mitochondrial Ca^2+^ overload and infarct size during cardiac ischemia-reperfusion [294]. In addition to the targets discussed above, there are many intracellular hot spots for ER-mitochondrial Ca^2+^ crosstalk that deserve further study. In conclusion, preclinical data using decoy or regulatory peptides acting on major Ca^2+^ channels in the ER-mitochondria will be needed to facilitate the rapid development of these tools into practical therapies.

## Figures and Tables

**Table 1:** Inhibitors and peptides that target mitochondrial calcium.

Name	Source/Mechanism	Proposed Mode of Action	References
CsA	an inhibitor of mPTP and can bind to CypD	a positive regulator of mPTP	[277,278,279]
mtCsA	non-immunosuppressive derivatives of CsA	desensitizes mPTP to Ca^2+^ and inhibits pore opening	[291]
BH4-Bcl-2	BH4 domain of Bcl-2	inhibit IP3R-mediated Ca^2+^ release	[280]
Bcl-2/IP3R disruptor 2 (Bird-2)	an IP3R-derived peptide	inhibit IP3R-mediated Ca^2+^ release	[280]
peptide A	a short fragment of the cytosolic II–III loop of the DHPR	induces RyR1-mediated Ca^2+^ release	[281]
Calcins	have a high affinity for RyR1 and specifically bind and stimulate its activity.	have similar effects on RyR1 gating	[283]
ANTP-N-TER	N-terminal of VDAC1	significantly inhibit mitochondrial Ca^2+^ uptake	[284]
ANTP-L14-15	short peptides from the N-terminal of LP4	inhibit mitochondrial Ca^2+^ uptake	[284]
R-tf-d-lp4 peptide	the VDAC1-based peptide	significantly increased intracellular Ca^2+^ levels	[285]
BH4-Bcl-XL	an inhibitor of RyR in ER	blocks VDAC1-mediated Ca^2+^ uptake into mitochondria	[286,287,288,289]
Nesiritide, Natrecor	recombinant BNP peptide	inhibiting MCU and affecting mPTP opening	[290]
Octapeptide RRNYRRNY (RNY)	inhibits the Cx43 hemichannels in mitochondria	reduces mitochondrial Ca^2+^ overload	[294]

## Data Availability

No new data were created or analyzed in this study. Data sharing is not applicable to this article.
